# Program Participation and Blood Pressure Improvement in the Heart of New Ulm Project, Minnesota, 2009–2011

**DOI:** 10.5888/pcd11.130205

**Published:** 2014-03-27

**Authors:** Arthur Sillah, Abbey C. Sidebottom, Jackie L. Boucher, Raquel Pereira, Jeffrey J. VanWormer

**Affiliations:** Author Affiliations: Abbey C. Sidebottom, Allina Health, Minneapolis, Minnesota; Jackie L. Boucher, Raquel Pereira, Minneapolis Heart Institute Foundation, Minneapolis, Minnesota; Jeffrey J. VanWormer, Epidemiology Research Center, Marshfield Clinic Research Foundation, Marshfield, Wisconsin.

## Abstract

**Introduction:**

The Heart of New Ulm (HONU) Project is a community-based heart disease prevention intervention that delivers various component programs through health care, work sites, and the community. We examined the association between HONU program participation and blood pressure (BP) control over the first 2 years of the project.

**Methods:**

The sample included residents aged 40 to 79 years from the target zip code who attended a heart health screening at baseline (2009) and again at follow-up (2011). BP control was defined as achieving or maintaining a BP less than 140/90 mm Hg in 2011.

**Results:**

BP improvements were observed in the sample: 81.7% of those who had controlled BP in 2009 maintained controlled BP 2 years later, and 52.4% of those with uncontrolled BP at baseline had controlled BP 2 years later (mean [SD] change in systolic BP, −10.6 mm Hg [20.8]). In the final adjusted model, participation in any 2 component programs of the HONU Project was associated with significantly higher odds of BP control among those with uncontrolled BP at baseline (n = 374). Participation in any component of the HONU Project among those with uncontrolled BP was associated with significant BP improvement compared with no participation.

**Conclusions:**

The clinical, work site, and community education and behavioral programs (eg, healthful diet or physical activity) delivered as part of a population-level heart disease prevention intervention were associated with meaningful BP improvements over 2 years among those with uncontrolled BP at baseline.

## Introduction

Cardiovascular disease (CVD) is the leading cause of death and disability among adults worldwide (1). Although the last few decades have seen a decrease in CVD mortality in the United States and many other countries (2), less progress has been made on reducing incident CVD in the upper midwestern United States (3). The rising prevalence of obesity and diabetes (4) coupled with higher prevalence of coronary heart disease (CHD) in rural areas (5) highlights the importance of reducing the burden of CVD risk factors in rural communities. High blood pressure (BP) is the most prevalent major CVD risk factor (nationally, 31% of men and 33% of women have high BP) (6) and shows few epidemiologic signs of abatement. High BP promotes atherogenesis, resulting in a twofold to threefold increase in CVD risk (7) and carries the highest population-attributable risk among CHD risk factors (8).

Descriptions of community-based initiatives designed to improve BP across a defined population are rare, particularly in rural areas. Only the Stanford Five-City project of 3 major US community-based CVD prevention studies achieved significant (4%) reductions in blood pressure over 5 years, mainly limited to women (9,10). More recently, a WISEWOMAN community screening intervention program was successful in reducing high BP by 7% and 9% in 2 intervention groups in Massachusetts (11).

In response to the gap in community-level CVD health improvement research, the Hearts Beat Back: The Heart of New Ulm (HONU) Project (www.heartsbeatback.org) was developed as a multi-year CVD prevention research and demonstration project serving rural New Ulm, Minnesota (12,13). The HONU Project implemented heart health screenings to assess risk factors in the target community. Various intervention programs were offered through the health care system, work sites, and the general community after baseline assessments. The objective of this analysis was to examine 2-year changes in BP in the target population and to assess the degree to which maintaining BP control among those with normal BP or achieving BP control among those with uncontrolled BP at baseline was associated with participation in HONU intervention programs (both numbers and types of programs).

## Methods

### Setting

New Ulm is about 100 miles southwest of the Minneapolis–St Paul, Minnesota, metropolitan area in an agricultural region of the state. The HONU Project, initiated in 2009, is a 10-year initiative primarily designed to target the approximately 7,900 adults aged 40 to 79 years who reside in the zip code that surrounds New Ulm (56073). The long-term goal of the project is to reduce acute myocardial infarction rates and the short-term goal is to reduce the prevalence of 9 modifiable CVD risk factors (12,13). The HONU Project is a collaborative partnership of Allina Health, the Minneapolis Heart Institute Foundation, and the community of New Ulm. New Ulm Medical Center, an Allina-owned hospital and clinic, is the primary health care service provider within the target population and a key organization in implementation of many HONU intervention programs.

The HONU Project is designed to implement evidence-informed health improvement practices, based on the community’s level of risk and preferences. To establish an understanding of the community’s level of risk, comprehensive heart health screenings, similar to those used in prior population-based prevention programs (14,15), were offered free to the community in 2009. These screenings served both as a needs assessment tool and as an intervention to educate residents on their risk factors and to offer health coaching around lifestyle changes and guidance for follow-up for managing medical conditions. Results from screenings identified high obesity rates, high prevalence of metabolic syndrome, low fruit and vegetable consumption, and low use of preventive medical therapies as interventions for priority risk factors (12,13).

HONU interventions are aligned with a social–ecological model of health determinants and health promotion addressing CVD risk factors at individual, social, institutional, community, and policy levels (16). Interventions are generally delivered through health care, work sites, and the general community by using best practices from previous interventions delivered in these contexts (17,18) (Appendix). Health care interventions include a health-care–based telephone coaching program for people at high risk for CVD, kiosks placed in the community to assess risk factors (ie, blood pressure and weight), and education of health care providers. Work site intervention programs targeted the community’s largest employers who were asked to implement wellness policies and health-related programs (eg, behavior change programs of 6 to 8 weeks focused on healthy eating and exercise for employees) and education programs for human resources managers on benefits planning, benefits design, and health promotion. Community-wide interventions include social marketing focused on heart health improvement; health challenges focused on increased physical activity, fruit and vegetable consumption, and weight management; educational programs delivered to the community through various venues (eg, a local cooking television show, educational grocery store tours, an electronic and print newsletter, a website with education and program activity resources); a volunteer neighborhood leader program focused on community organizing around increased local health promotion activities; and environmental re-engineering efforts in the food environment (12,13).

### Screening procedures

The heart health screening program was free to all people 18 years or older who presented in person at screening sites (eg, work sites, community centers, churches). As described in more detail elsewhere (12,13), the 2009 screenings were promoted to residents through letters, advertisements, work sites, and by health care providers at New Ulm Medical Center. Screenings ran from mid-April to mid-December 2009. The screening process was repeated in 2011 using similar methods. The Allina Institutional Review Board approved all procedures for the screenings and approved use of screening data for this study.

Screening participants were asked to fast 12 hours before their appointment. Screening lasted 20 to 30 minutes and included registration and consent, health history and behavioral risk factor survey, anthropometric measures (ie, height, weight, waist circumference, and blood pressure), and venipuncture. Participants were given a personal risk factor report and met with a health coach (ie, registered dietitian or health educator) to review risk factors, discuss health improvement goals, and get guidance on community resources or other health education opportunities and referrals for any immediate medical follow-up.

### Design and measures

This analysis used a longitudinal panel design. Data on people who were screened for CVD risk factors in both 2009 and 2011 were included in the analysis. The primary outcome was BP in 2011, which was categorized as controlled (<140/90 mm Hg) or uncontrolled (≥140/90 mm Hg) based on the Fifth Report of the Joint National Committee (JNC) on Detection, Evaluation, and Treatment of High Blood Pressure (19) and in alignment with criteria used in other population studies (20,21). Although the Seventh JNC Report recommends a lower goal (130/80 mm Hg) for high-risk people such as patients with diabetes (22), a study among people with diabetes found no difference in outcomes based on the lower BP goal compared with a goal of less than 140/90 mm Hg (23).

Trained staff followed an adaptation of the Canadian Hypertension Society guidelines (24). They measured each participant’s BP using a SunTech 247 device (SunTech Medical, Morrisville, North Carolina) after sitting for 3 minutes. Three BP values were measured, taken 1 minute apart, using an automatic sphygmomanometer. The mean of the last 2 BP measures was used for analytical purposes.

The main predictor was participation in a HONU intervention program between 2009 and 2011. Similar to methods used in previous large community CVD prevention projects (14), program participation was assessed by self-report during the 2011 screening with a single item where participants indicated which of 12 programs they participated in over the previous 2 years. Program participation was operationalized using 2 independent methods. First, participation was grouped into 5 categories based on program focus and delivery. These were 1) education — read at least 1 HONU newsletter (print or e-mail) or visited the HONU website; 2) physical activity — participated in the community health challenge or neighborhood walking program; 3) healthy eating — participated in at least 1 neighborhood potluck, supermarket tour, or cooking class, or watched at least 1 episode of the HONU healthy cooking television show; 4) clinical — participated in the telephone coaching program or visited a heart health station at 1 of 4 local sites; and 5) work site — participated in at least 1 work site wellness program. Second, to estimate total program exposure, the number of programs participated in by each participant was summed. Several baseline covariates were also included in analytical models based on their previously known or clinically suspected association with BP and program participation. These included age, sex, education level, smoking status, body mass index (BMI), personal history of diabetes or heart disease, and antihypertensive medication use at baseline.

### Analysis

All analytical procedures were conducted using SAS (PC SAS 9.2, SAS Institute Inc, Cary, North Carolina). Means and standard deviations for continuous variables and percentages for categorical variables were described. Paired *t* tests and Bowker’s test of symmetry were used to assess changes in BP and medication use between 2009 and 2011. Multivariable logistic regression (PROC LOGISTIC) was used to examine the association between BP and program participation (modeled separately by program type as well as number of programs participated in). To gauge BP improvement versus maintenance of controlled BP, the analytical sample was stratified by those with controlled and uncontrolled baseline BP. Identical procedures were used for both analyses. First, a basic model was created to examine the crude relationship between program participation and BP. Then a full model was created with all covariate terms entered simultaneously. Screening participants who did not participate in any program was the comparison group for the analysis.

## Results

A total of 3,123 participants in the target population were screened in 2009, of whom 1,455 (47%) returned for screening in 2011. Nine participants had missing BP data in 2011 and were excluded from further analyses. The final study sample of 1,446 represents 18% of all target area residents aged 40 to 79 years, per 2010 US Census estimates (25). Compared with those who did not return for screening in 2011, participants screened in both 2009 and 2011 were more likely to be women (63% vs 53.4%, *P* < .001) and were less likely to be obese (38% vs 43.3%, *P* < .001) or have high BP (25.9% vs 29.6%, *P* < .001). In addition, 96% were white, 35% had college or higher education, 99% were insured, and they had an average age (SD) of 56.3 (13.6) years.

### Program participation

The educational programs had the greatest reach as 56.1% of participants had read at least 1 HONU newsletter and 41.7% visited the HONU website (Table 1). Activities that included time commitments over several weeks such as the community health challenge and work site wellness programs had similar levels of participation (17%). Grocery store tours, cooking classes, and neighborhood programs had lower levels of participation with less than 10% participation rates in each. The controlled and uncontrolled baseline BP groups showed similar participation trends in 1, 2, or 3 or more programs. Overall, 77.5% of the study sample participated in at least 1 program. Program participants were more likely to have college or higher education (37% vs 29%, *P* = .01), to be women (69% vs 42.4%, *P* < .001), and less likely to be obese (36% vs 45.1%, *P* = .003) or to have a self-reported history of heart disease or diabetes (9.25% vs 14.2%, *P* = .01) at baseline compared with the 22% who did not participate in any program.

**Table 1:** Number and Category of Component Programs Used by Screened Participants Aged 40 to 79 Years, Stratified by Baseline Blood Pressure Status, Heart of New Ulm Project, Minnesota, 2009–2011

Program Category/ Individual Program Components	Total (N = 1,446), %	Baseline Controlled Blood Pressure (n = 1,072; 74.1%)			Baseline Uncontrolled Blood Pressure (n = 374; 25.9%)		
		1 Program (n = 203), %	2 Programs (n = 234), %	≥3 Programs (n = 401), %	1 Program (n = 75), %	2 Programs (n =77), %	≥3 Programs (n = 130), %
**Healthy eating programs**							
Neighborhood healthy potluck	1.7	0.5	0.9	3.7	0	1.3	3.9
Cooking class	6.2	2.0	1.7	14.5	2.7	1.3	15.4
What’s Cooking New Ulm television show	21.6	8.4	21.4	38.2	8.0	23.4	52.3
Grocery store tour	2.0	1.0	0.4	4.7	0.0	0.0	5.4
**Physical activity programs**							
Neighborhood walking club	4.9	1.5	1.7	13.7	0.0	1.3	6.2
Neighborhood lifestyle program	8.9	0.0	3.9	11.5	2.0	5.1	23.4
Health challenge	16.9	4.4	13.7	37.2	1.8	6.5	37.7
**General education**							
Visited website	41.7	17.2	47.0	80.1	6.7	42.9	76.2
HONU e-newsletter	56.1	42.4	68.8	88.5	52.0	70.3	89.2
**Clinical program**							
Telephone coaching program	11.4	7.9	9.0	18.2	14.7	11.7	26.9
Heart health station	18.0	4.9	18.0	31.9	8.0	19.5	45.4
**Work site**							
Work site wellness program	17.1	7.9	12.4	36.7	6.7	18.2	27.7
**Overall participation in at least 1 program**	77.5	78.2			75.4		

### Blood pressure

During the 2009 baseline period, mean (SD) systolic/diastolic BP across the analytical sample was 127.8 (16.7)/76.5 (9.9) mm Hg, and 25.9% of participants had uncontrolled BP. Antihypertensive medication was reported by 28.4% of participants at baseline. By the 2011 follow-up, systolic BP dropped by 2.9 (14.9) mm Hg, along with a 2.5 (8.5) mm Hg drop in diastolic BP (*P* < .001 for all changes). BP improvements were most pronounced among those with uncontrolled BP at baseline. Systolic and diastolic BP were reduced by 10.6 (20.8) and 6.5 (10.9) mm Hg (approximately 7%), respectively, with 52.4% of those with uncontrolled BP at baseline achieving BP control. Among those with controlled BP at baseline, 81.7% maintained their controlled BP status by 2011, with stable systolic BP and a small decrease of 1.1 (6.9) mm Hg in diastolic BP (Table 2).

**Table 2:** Blood Pressure and Medication Use at Baseline Screening in 2009 and Change From Baseline in 2011 for Participants Aged 40 to 79 Years, The Heart of New Ulm Project, Minnesota

Status	Total, (N = 1,446)			Controlled Baseline Blood Pressure, (n = 1,072; 74.1%)			Uncontrolled Baseline Blood Pressure, (n = 374; 25.9%)		
	Baseline	Change From Baseline	*P* Value^a^	Baseline	Change From Baseline	*P* Value^a^	Baseline	Change From Baseline	*P* Value^a^
Hypertension medication use, %	28.4	5.4	<.001	23.6	2.2	<.001	42.3	14.4	<.001
Systolic blood pressure, mmHg, mean (SD)	127.8 (16.7)	−2.9 (14.9)	<.001	121.0 (10.5)	−0.2 (11.0)	.57	147.3 (15.9)	−10.6 (20.8)	<.001
Diastolic blood pressure, mmHg, mean (SD)	76.5 ( 9.9)	−2.5 (8.5)	<.001	74.2 (8.5)	−1.1 (6.9)	<.001	82.9 (10.8)	−6.5 (10.9)	<.001
Controlled blood pressure, %^b^	NA			81.7			52.4		

Abbreviation: NA, not applicable.

a *P* values are obtained from paired *t* test for continuous measures and categorical measures from Bowker’s test of symmetry.

b This is the proportion of those with controlled blood pressure at baseline that had maintained control of blood pressure in 2011 and the proportion of people who improved to controlled blood pressure among the group with uncontrolled blood pressure at baseline.

Those with uncontrolled BP at baseline were also more likely to report use of antihypertensive medication (42.3% vs 23.6% among those with controlled BP at baseline) (Table 2). The group with uncontrolled BP at baseline also experienced a larger increase in the use of medications between the 2 screenings.

Multivariable regression modeling found, among those with controlled BP in 2009, no significant association between the number of programs that were participated in and BP control in 2011 (Table 3). Similarly, no significant association was found between program type and BP control in 2011 among those with controlled BP at baseline (Table 3). However, among those with uncontrolled BP in 2009, there was a significantly greater adjusted odds of controlled BP in 2011 for those who participated in 2 programs (odds ratio [OR], 2.45; 95% confidence interval [CI], 1.28–4.68) versus none. Furthermore, compared with those who did not participate in any program, participation in general educational (OR, 2.26; 95% CI, 1.32–3.85), clinical (OR, 2.60; 95% CI, 1.44–4.70), physical activity (OR, 2.70; 95% CI, 1.36–5.37), healthy eating (OR, 1.94; 95% CI, 1.05–3.60) or work site (OR, 2.79; 95% CI, 1.33–5.85) programs showed an increased odds of BP improvement in 2011 (Table 3). Blood pressure medication use at baseline was not a significant covariate in any model.

**Table 3:** Multivariable Association Between Program Participation, Type of Program, and Controlled Blood Pressure Among Participants Aged 40 to 79 Years, Stratified by Baseline Blood Pressure Status, Heart of New Ulm Project, Minnesota, 2009–2011

Program Participation/Model	Baseline Controlled Blood Pressure (n = 1,072; 74.1%)		Baseline Uncontrolled Blood Pressure (n = 374; 25.1%)	
	OR (95% CI)^a^	*P* Value^b^	OR (95% CI)^a^	*P* Value^b^
**Program participation by number of programs (reference category is “no program participation”)**				
**Model 1**				
1 program	1.06 (0.73–1.56)	.75	1.24 (0.75–2.06)	.41
2 programs	0.93 (0.59–1.46)	.75	2.45 (1.28–4.68)	.01
≥3 programs	1.65 (0.92–2.99)	.09	1.54 (0.76–3.12)	.23
**Program participation by program type (reference category is “no program participation”)**				
**Model 2**				
General education	1.19 (0.79–1.77)	.41	2.26 (1.32–3.85)	.002
Other types of programs	1.11 (0.64–1.93)	.72	2.12 (1.03–4.40)	.04
**Model 3**				
Healthy eating program	1.29 (0.81–2.07)	.29	1.94 (1.05–3.60)	.03
Other types of programs	1.12 (0.74–1.68)	.60	2.40 (1.39–4.13)	.002
**Model 4**				
Physical activity programs	1.47 (0.90–2.41)	.12	2.70 (1.36–5.37)	.004
Other types of programs	1.07 (0.72–1.60)	.74	2.12 (1.25–3.60)	.01
**Model 5**				
Clinical program	1.12 (0.70–1.78)	.64	2.60 (1.44–4.70)	.002
Other types of programs	1.20 (0.79–1.81)	.39	1.99 (1.14–3.48)	.02
**Model 6**				
Work site programs	1.07 (0.64–1.79)	.80	2.79 (1.33–5.85)	.01
Other types of programs	1.20 (0.80–1.79)	.37	2.13 (1.25–3.61)	.01

Abbreviations: OR, odds ratio; CI, confidence interval.

a OR adjusted for baseline (2009) educational level, age, body mass index, disease history (diabetes or heart), blood pressure medication use, smoking, and sex in each model.

b *P* values are Wald χ^2^ statistics from the logistic regression for number of program categories and component programs predicting blood pressure control in 2011.

## Discussion

The trends in improved BP control over 2 years were encouraging in this sample, particularly among those with uncontrolled BP at baseline where systolic BP decreased by over 10 mm Hg and antihypertensive medication use increased by 14%. This level of improvement over 2 years was roughly equivalent to that observed in the US population over the previous 10 years (26). On the basis of pooled systolic BP and mortality associations from 5 large longitudinal population studies (27), an approximately 10 mm Hg reduction in systolic BP among those with high baseline BP would be expected to reduce CHD mortality by nearly 20% and stroke by nearly 30%. These findings are generally consistent with preliminary HONU Project analyses that have observed reduced occurrence of myocardial infarctions in the area (28).

The benefits of program participation were mixed. Among those with controlled baseline BP, program participation had little influence on the maintenance of BP control. Among those with uncontrolled baseline BP, however, participation in any program approximately doubled the odds of controlled BP over 2 years, with those who participated in 2 programs having the greatest chance of controlled BP relative to nonparticipants. All program types were beneficial for this group.

These findings create several implications for program design in the context of community-level CVD prevention initiatives. The benefits observed for those with uncontrolled baseline BP were least surprising because this group had the greatest near-term incentive to make health improvements. The fact that all program types were beneficial, however, was unexpected given the varying degrees of intensity in each, ranging from low-intensity programs (eg, newsletters and website) in general education programs to more intense didactic interactions with telephone coaches in the clinical program or short-term behavioral change programs delivered through work sites or the community. Explanations for this collective program success are unclear, but based on the general self-reported increase in antihypertensive medication use over time, program participants may have somehow experienced greater opportunities or openness to address BP control during medical encounters. The potential effect of the HONU Project on primordial hypertension control seems limited. BP was stable over 2 years in those with controlled baseline BP, and this finding was independent of direct program participation. Widespread participation in the neighborhood physical activity and healthy eating programs was not observed, but plans are under way to increase their reach because they have the potential to attract a broad cross-section of the population (including families with children) in other facets of CVD prevention (eg, lifestyle change).

There are several implications of these findings for health care providers in rural communities. First, health care organizations can be a lead partner for any prevention activities even if implemented through other venues such as work sites or for the general public. Provider referral for these programs may be an important part of recruitment and retention. Additionally, the health care components may improve providers’ ability to address prevention through their clinical practice. For example, screening data on behavioral risk factors such as poor nutrition and lack of exercise can complement existing health record data since systematic measures of these patient-related factors are not typically available to providers. Providers can use this information to better counsel patients on these health risks, track changes, and tailor treatment.

The chief methodologic limitation of this study involved healthy volunteer bias, which is rarely accounted for in community-level research. The analytical sample in this study was composed of data on a subgroup of participants who were screened in both 2009 and 2011. However, our study participants represented about half of all screened participants and just 18% of the entire target population. Because those available for follow-up were generally healthier than those unavailable, the observed results may have limited generalizability to the entire community (ie, participants more inclined to get rescreened may have had a greater underlying propensity to benefit from the HONU Project). This hypothesis is somewhat supported by previous findings that people who are physically active and who have few lifestyle risk factors are more likely to be satisfied with preventive health care services (and are therefore more likely to participate in screenings or other such activities) (29). Self-reported program participation measures were also a limitation, but the objective measurement of BP was a strength, which was assessed several times at each screening visit by a trained professional using a calibrated SunTech 247 device in both years (30).

Among those with high BP at baseline, BP improved over 2 years, and this outcome was at least partially attributable to integrated prevention programs. Various program types seemed to benefit those with uncontrolled baseline BP, whereas most participants with controlled baseline BP had stable BP over the 2 years regardless of program participation. Further research is needed to determine the optimal suite of work site, clinical, and community-oriented programs that will have the greatest effect on primary and secondary CVD prevention.

## Appendix. Heart of New Ulm (HONU) Project Intervention Programs Delivered Through the Community, Work Sites, and Health Care Programs, 2009–2011

**Table:**

Intervention	Implementation and Participation	Social Ecological Model Level	Intensity	Staff Delivering Interventions
**Community interventions**				
Heart health screenings	Assess heart disease risk factors: survey, anthropometric data, venipuncture, reporting, coaching. Educate and coach individuals on their risk level and health behaviors. Held at work sites and public community locations over 6 months (2009 and 2011).	Individual and institutional (work sites)	30-minute commitment	Medical center staff, registration staff, dietitians, health educators, community project manager, operations and community organizing staff
Community health summits	Annual community-wide inspirational events focused on lifestyle changes.	Individual and community	1 day	Community project manager, operations staff, guest speakers
Formal run/walk events	5 run/walk events (5K and 10K)	Individual and community	1 day for individuals for actual event plus training	Program operations and community organizing staff with community organizations
Community health challenges	6 total health challenges offered to the community following broad campaign themes encouraging small changes in physical activity, nutrition, and stress management. Individual participation using program materials and emails.	Individual and community	6–8 weeks, participation varied by individual	Community project manager, program operations staff, and work site manager
General education	Cooking classes, grocery store tours, and presentations. “What’s Cooking New Ulm TV Show” is presented on local cable access 7 times per week with 64 new episodes in 2010 and 2011.	Individual	Television show is weekly; grocery store tours last 1 hour; cooking classes vary	Dietitians and chefs, health educators
Small community events	New Ulm divided into 25 districts with trained volunteer leaders who promote opportunities for exercise and healthy events such as a physical activity class, walking clubs, healthy potluck, or dance-a-thon.	Individual and interpersonal	Varies	Community volunteers coordinated by community organization staff
Food environment improvement	Works with restaurants, grocery stores and convenience stores to improve healthy options available and promote those options.	Institutional and community	Varies by intervention site and level of program	Nutrition environment project manager, operations staff, dietitians
**Health care interventions**				
HeartBeat Connections	Telephone coaching program targeting patients at high cardiometabolic risk but without coronary heart disease. Goals: improve use of preventive medications and lifestyle-related risks. Integrated with primary care.	Individual	Varies by individual (calls occur about every 4–6 weeks and last 15–30 minutes)	Dietitians, nurses, health care program manager
Grand rounds	9 HONU grand rounds educational events conducted for physicians and mid-level providers.	Institutional (medical center) and individual (providers)	1–2 hours per event	Health care program manager, operations staff, guest speakers
**Work site interventions**				
Conducted Wellness Council of America (WELCOA) assessments	Assessment of 46 local business wellness policies and environment. Results included recommendations to improve their work site wellness programs and policies.	Institutional/policy	1 session for assessment and 1 or more sessions for results and recommendation implementation	Work site program manager
Heart health screenings conducted at work sites	29 companies. Reports given to each work site showing prevalence of risk factors among employees with recommendations for wellness programming targeting those risks.	Individual and institutional	30 minutes per participant, meetings with company leadership	Work site program manager and all staff needed for screenings
Work site behavioral change programs	Behavior change programs focused on weight loss, nutrition, or physical activity. 8 programs at 30 work sites.	Individual, interpersonal (teams), and institutional	6-8 weeks — level of individual or group activity varies by program	Work site program manager, operations staff
Business leader engagement and education	Annual employer summits with nationally recognized speakers, attended by 23–26 companies. Five educational events offered through the chamber of commerce; attendance, 34–36 companies.	Institutional	Varies depending on meeting (2 hours for summit, 1 hour for other events)	Work site program manager
